# Supplementary figures and images for: Saliency detection of textured 3D models based on multi-view information and texel descriptor
Source: PeerJ Comput Sci. 2023 Oct 25;9:e1584. doi: 10.7717/peerj-cs.1584 (PMC10703070; doi:10.7717/peerj-cs.1584)

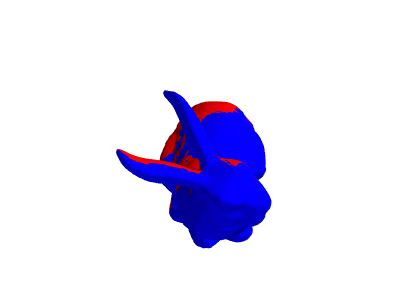

Supplement: Supplemental Information 1 [file peerj-cs-09-1584-s001.zip › py_2dto3d/image_saliency/snapshot1.png]

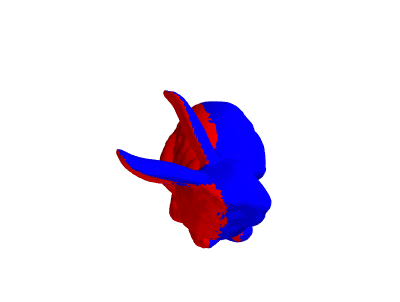

Supplement: Supplemental Information 1 [file peerj-cs-09-1584-s001.zip › py_2dto3d/image_saliency/snapshot2.png]

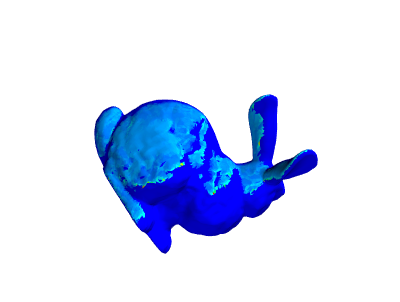

Supplement: Supplemental Information 1 [file peerj-cs-09-1584-s001.zip › py_2dto3d/image_saliency/snapshot3.png]

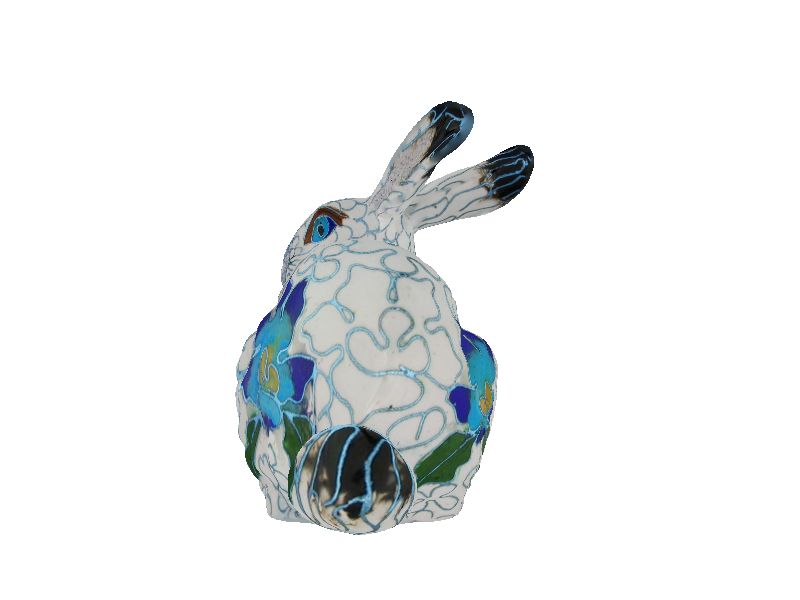

Supplement: Supplemental Information 1 [file peerj-cs-09-1584-s001.zip › py_2dto3d/out/+x0.png]

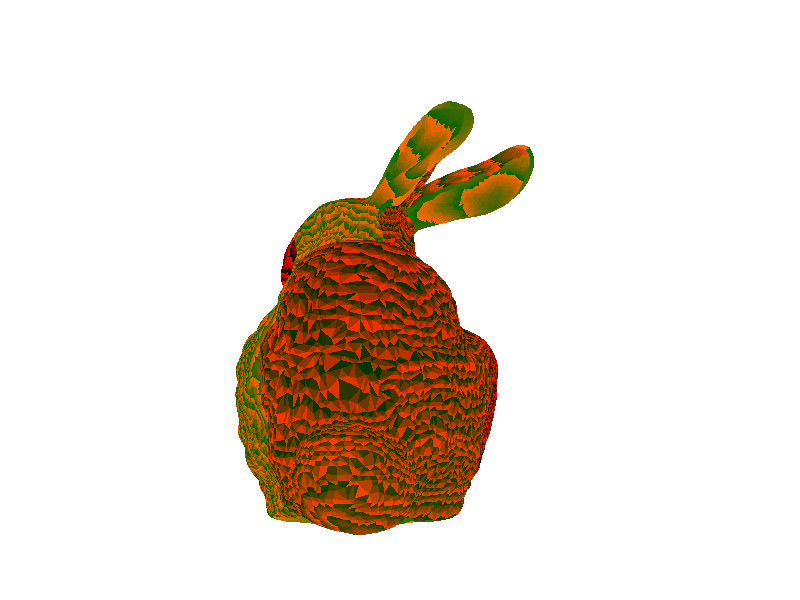

Supplement: Supplemental Information 1 [file peerj-cs-09-1584-s001.zip › py_2dto3d/out/+x1.png]

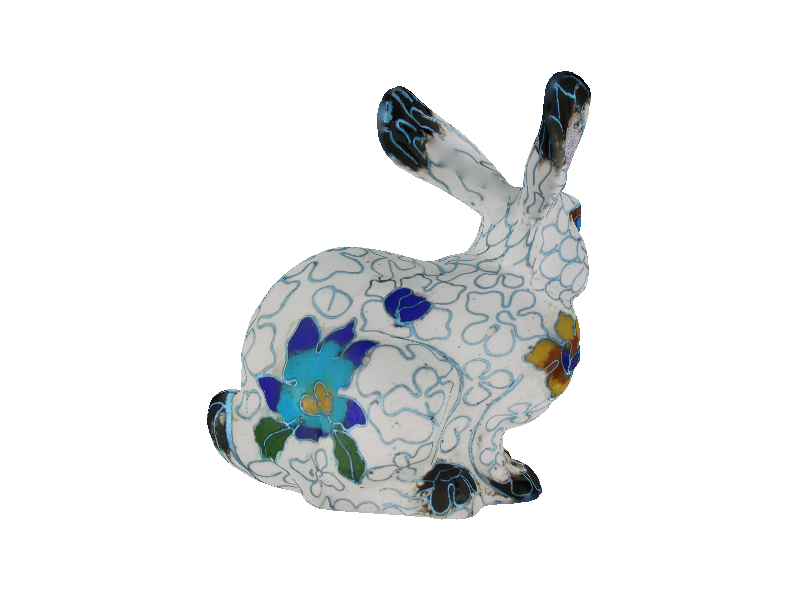

Supplement: Supplemental Information 1 [file peerj-cs-09-1584-s001.zip › py_2dto3d/out/+y0.png]

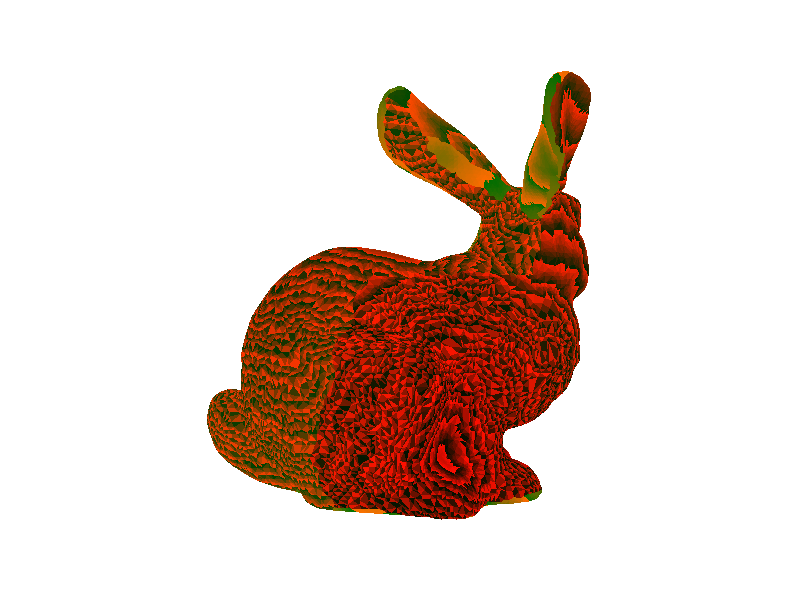

Supplement: Supplemental Information 1 [file peerj-cs-09-1584-s001.zip › py_2dto3d/out/+y1.png]

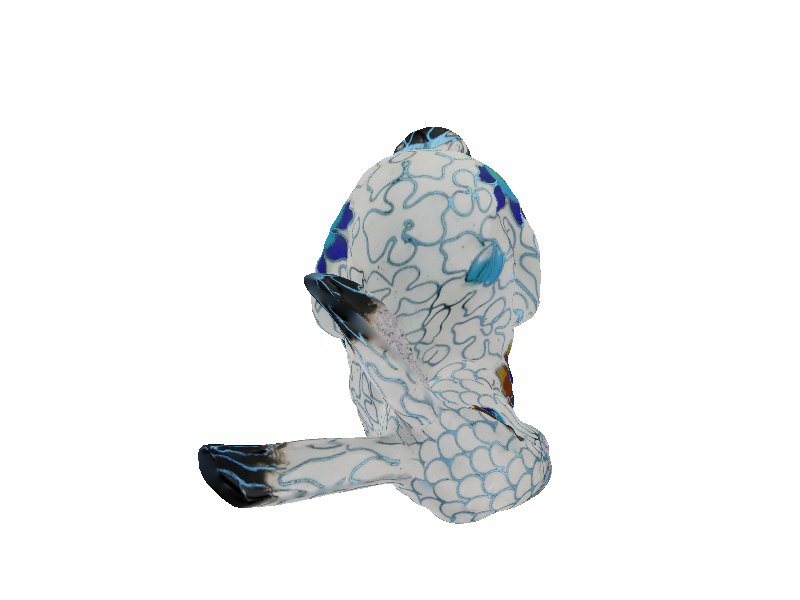

Supplement: Supplemental Information 1 [file peerj-cs-09-1584-s001.zip › py_2dto3d/out/+z0.png]

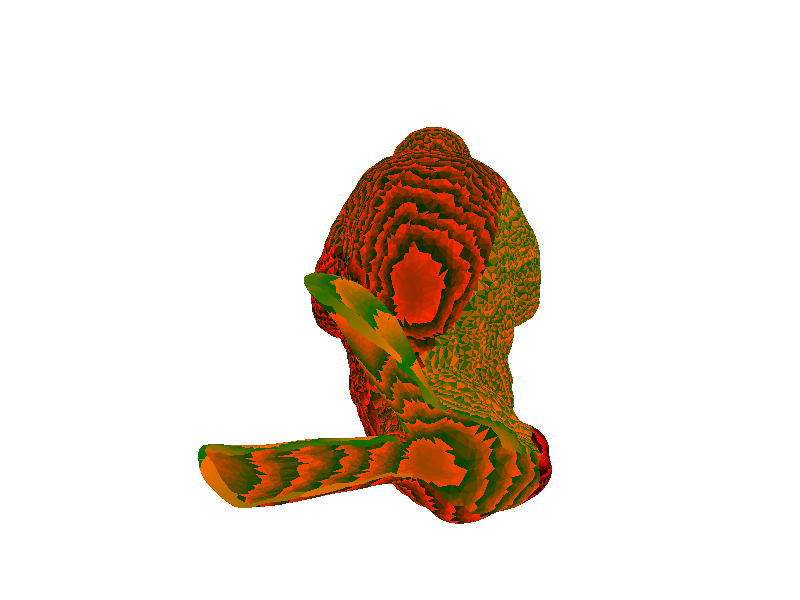

Supplement: Supplemental Information 1 [file peerj-cs-09-1584-s001.zip › py_2dto3d/out/+z1.png]

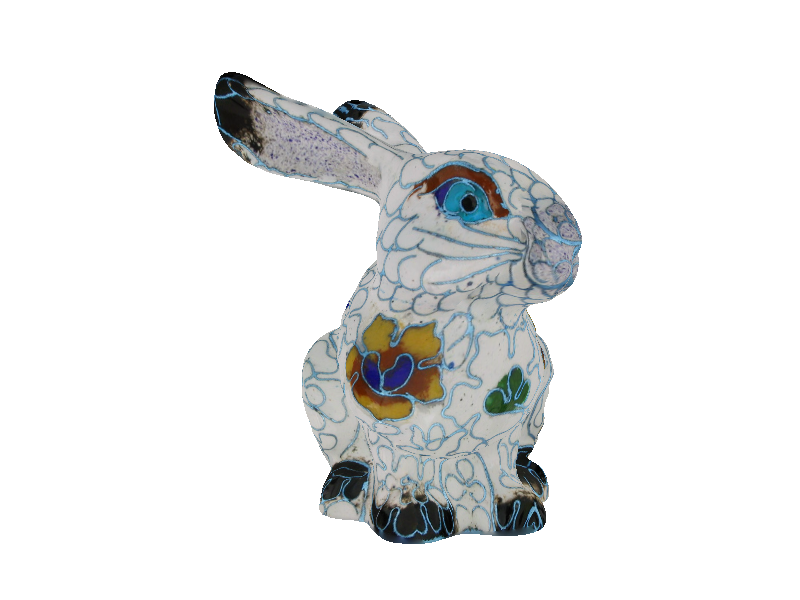

Supplement: Supplemental Information 1 [file peerj-cs-09-1584-s001.zip › py_2dto3d/out/-x0.png]

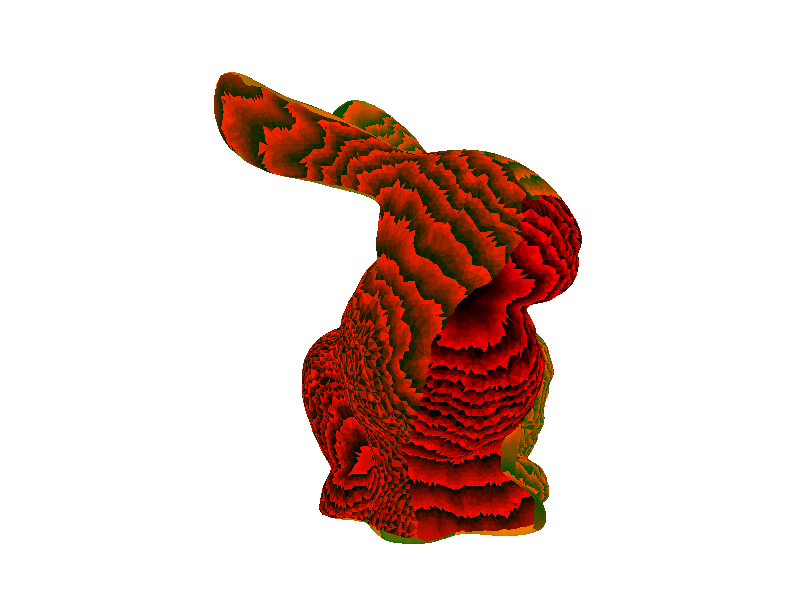

Supplement: Supplemental Information 1 [file peerj-cs-09-1584-s001.zip › py_2dto3d/out/-x1.png]

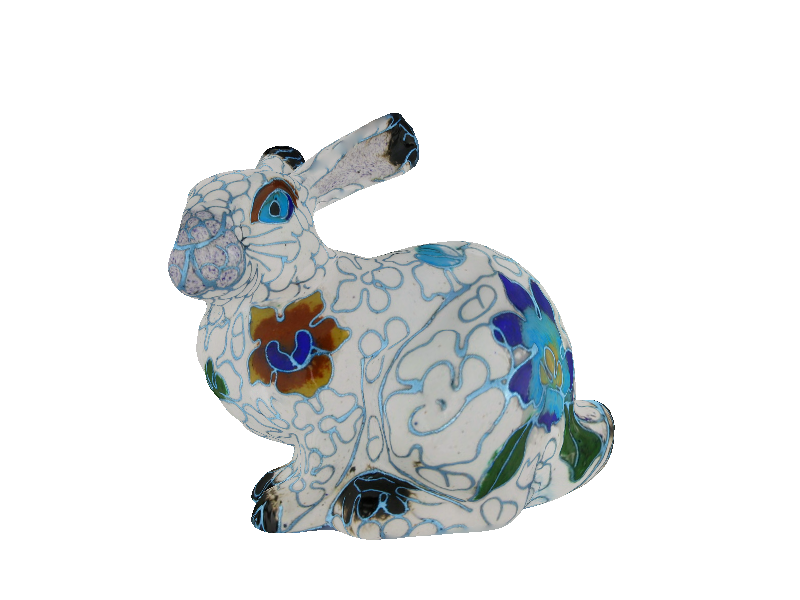

Supplement: Supplemental Information 1 [file peerj-cs-09-1584-s001.zip › py_2dto3d/out/-y0.png]

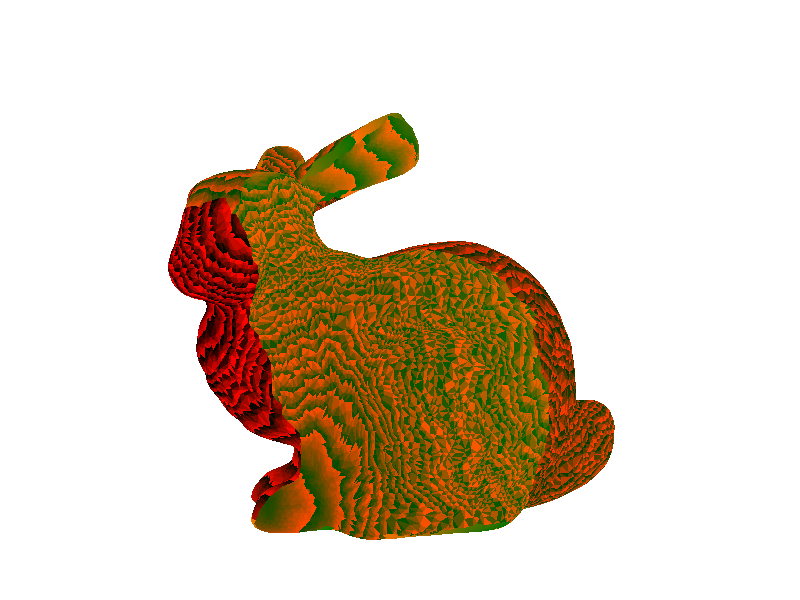

Supplement: Supplemental Information 1 [file peerj-cs-09-1584-s001.zip › py_2dto3d/out/-y1.png]

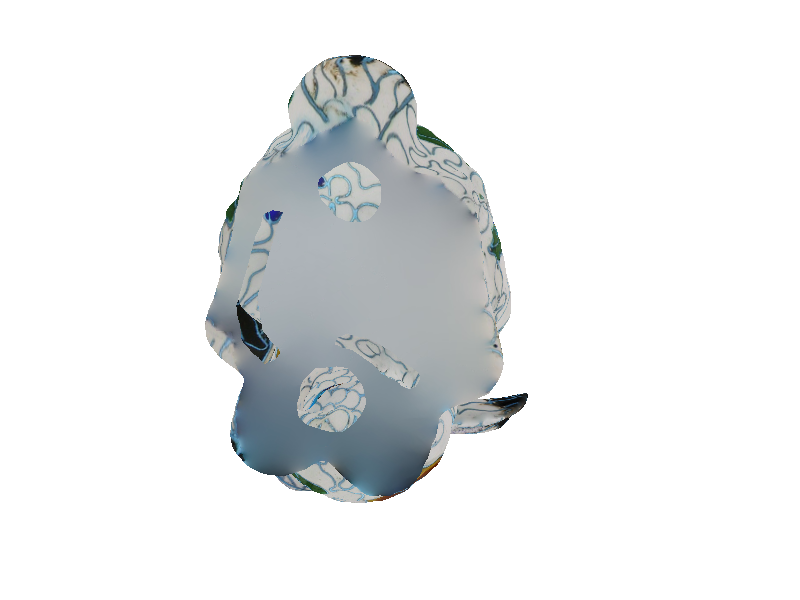

Supplement: Supplemental Information 1 [file peerj-cs-09-1584-s001.zip › py_2dto3d/out/-z0.png]

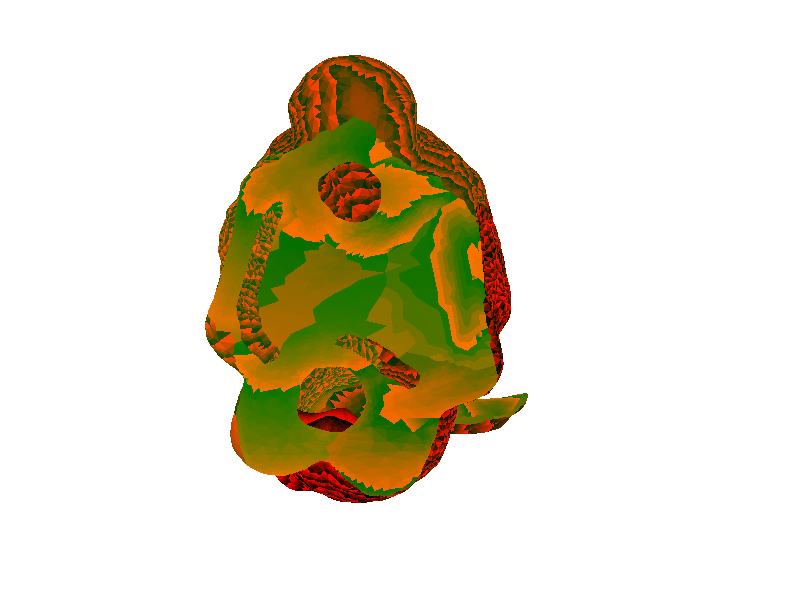

Supplement: Supplemental Information 1 [file peerj-cs-09-1584-s001.zip › py_2dto3d/out/-z1.png]

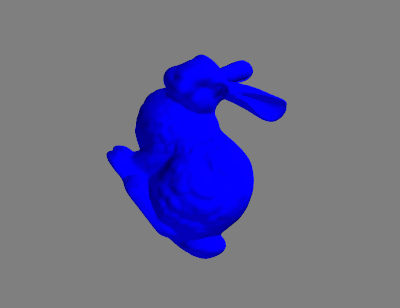

Supplement: Supplemental Information 1 [file peerj-cs-09-1584-s001.zip › py_2dto3d/out2/x1.png]

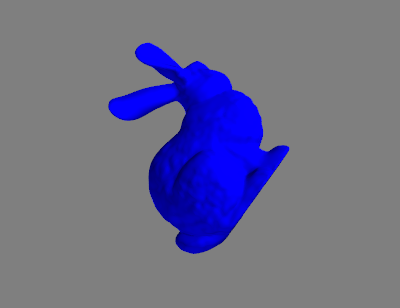

Supplement: Supplemental Information 1 [file peerj-cs-09-1584-s001.zip › py_2dto3d/out2/x2.png]

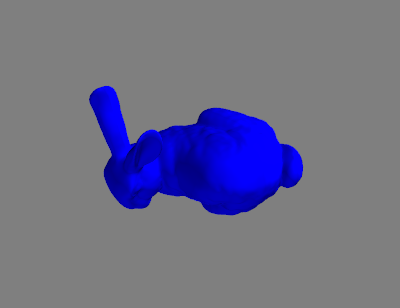

Supplement: Supplemental Information 1 [file peerj-cs-09-1584-s001.zip › py_2dto3d/out2/y1.png]

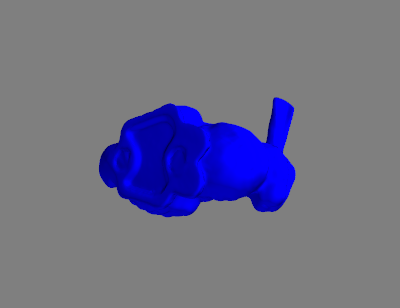

Supplement: Supplemental Information 1 [file peerj-cs-09-1584-s001.zip › py_2dto3d/out2/y2.png]

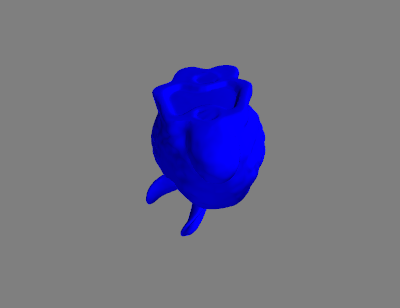

Supplement: Supplemental Information 1 [file peerj-cs-09-1584-s001.zip › py_2dto3d/out2/z1.png]

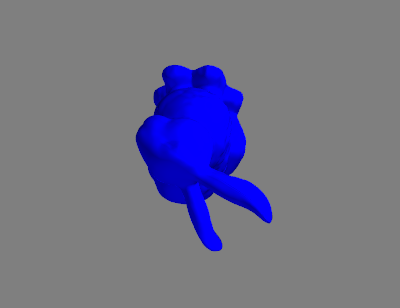

Supplement: Supplemental Information 1 [file peerj-cs-09-1584-s001.zip › py_2dto3d/out2/z2.png]

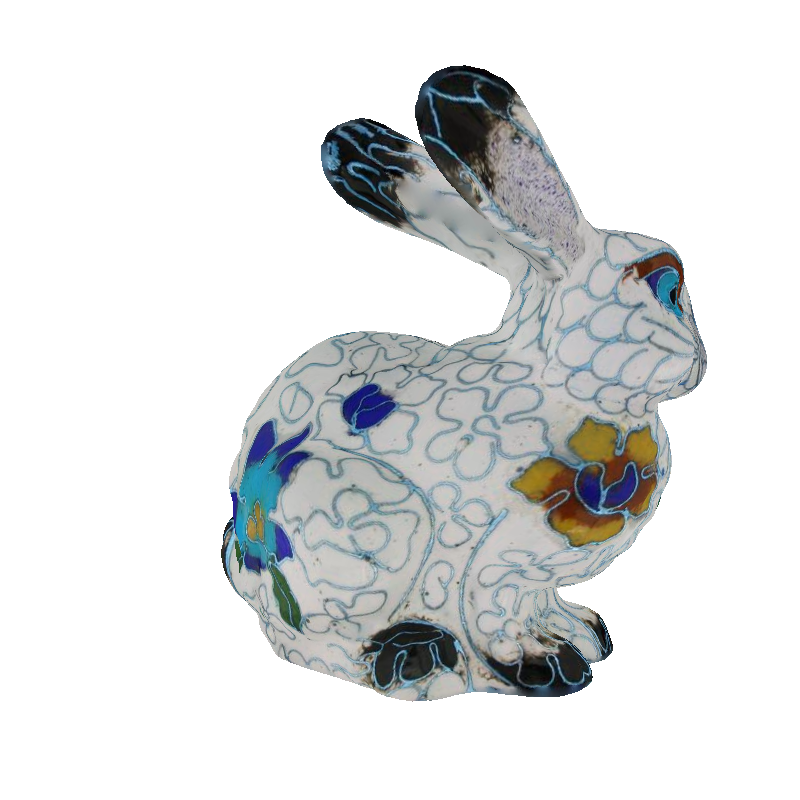

Supplement: Supplemental Information 1 [file peerj-cs-09-1584-s001.zip › py_2dto3d/video0/125.png]

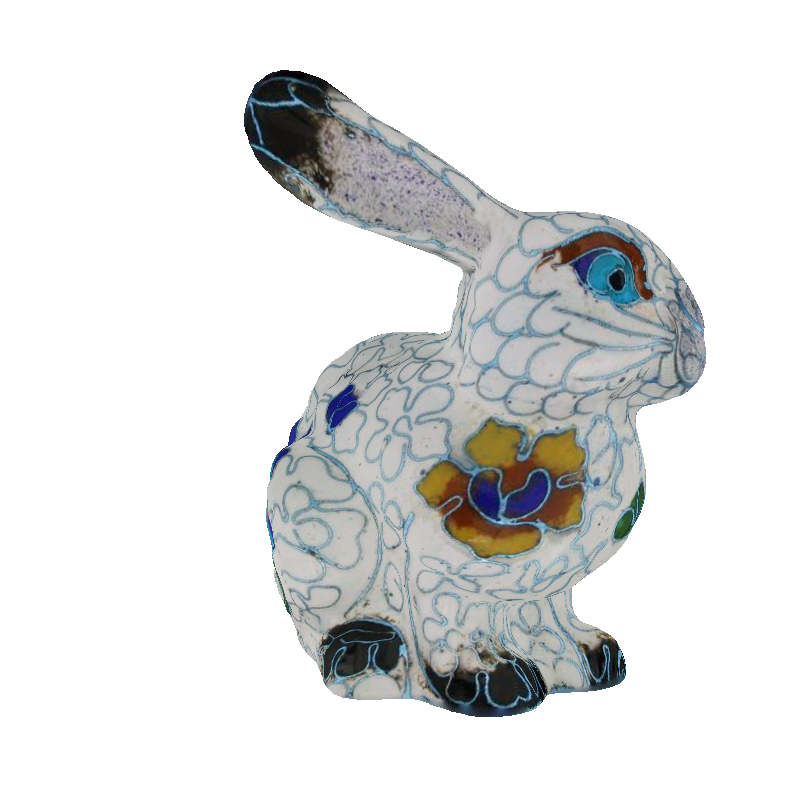

Supplement: Supplemental Information 1 [file peerj-cs-09-1584-s001.zip › py_2dto3d/video0/155.png]

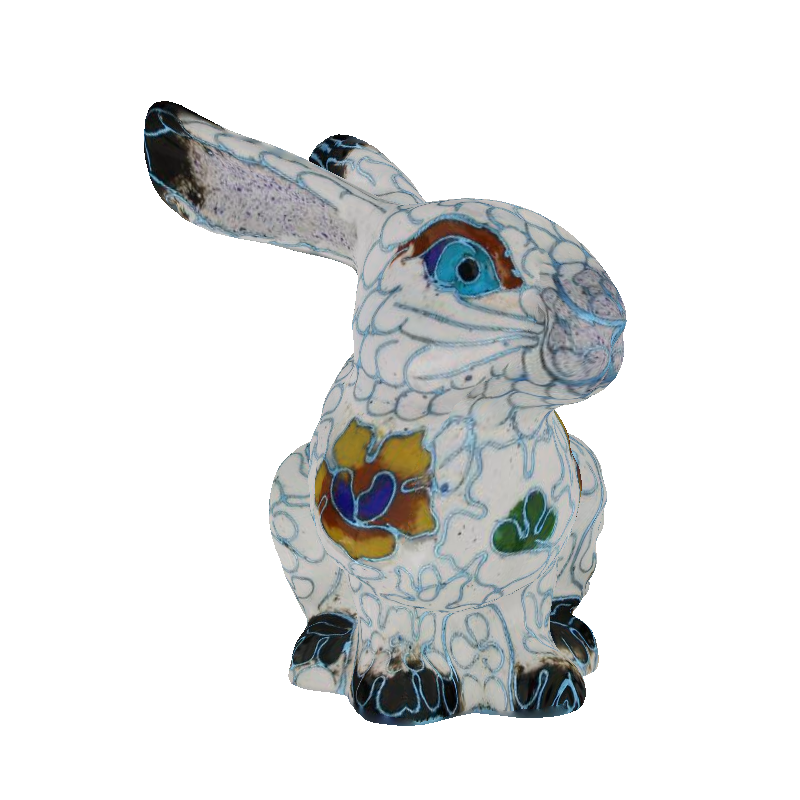

Supplement: Supplemental Information 1 [file peerj-cs-09-1584-s001.zip › py_2dto3d/video0/185.png]

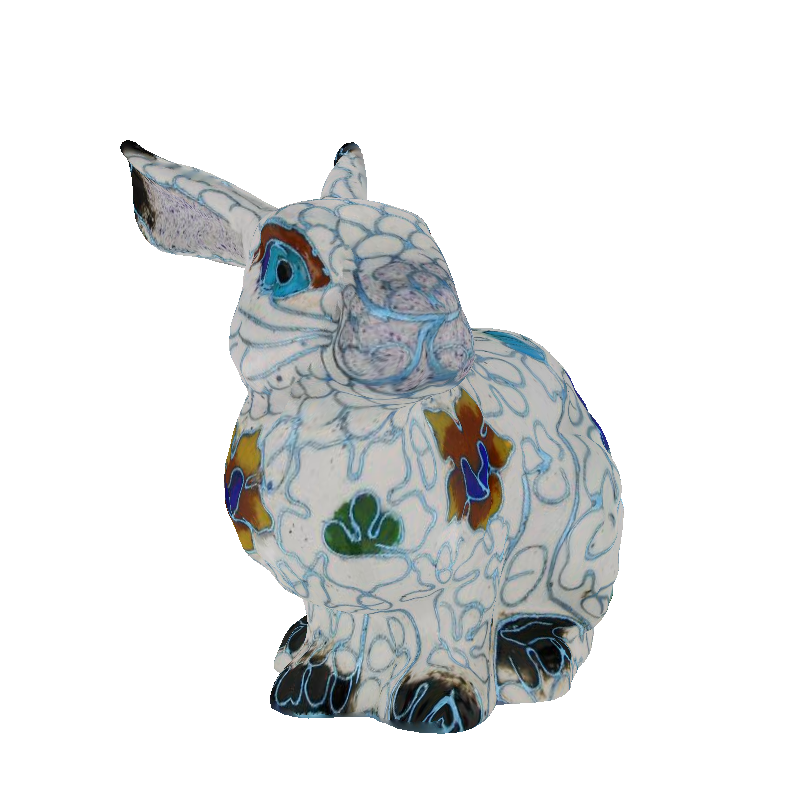

Supplement: Supplemental Information 1 [file peerj-cs-09-1584-s001.zip › py_2dto3d/video0/215.png]

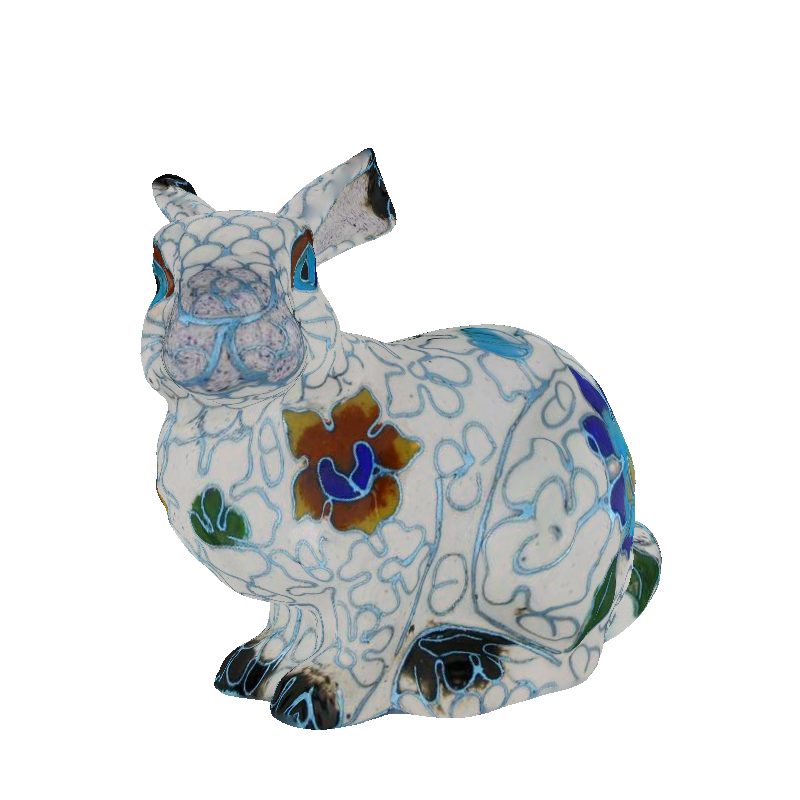

Supplement: Supplemental Information 1 [file peerj-cs-09-1584-s001.zip › py_2dto3d/video0/245.png]

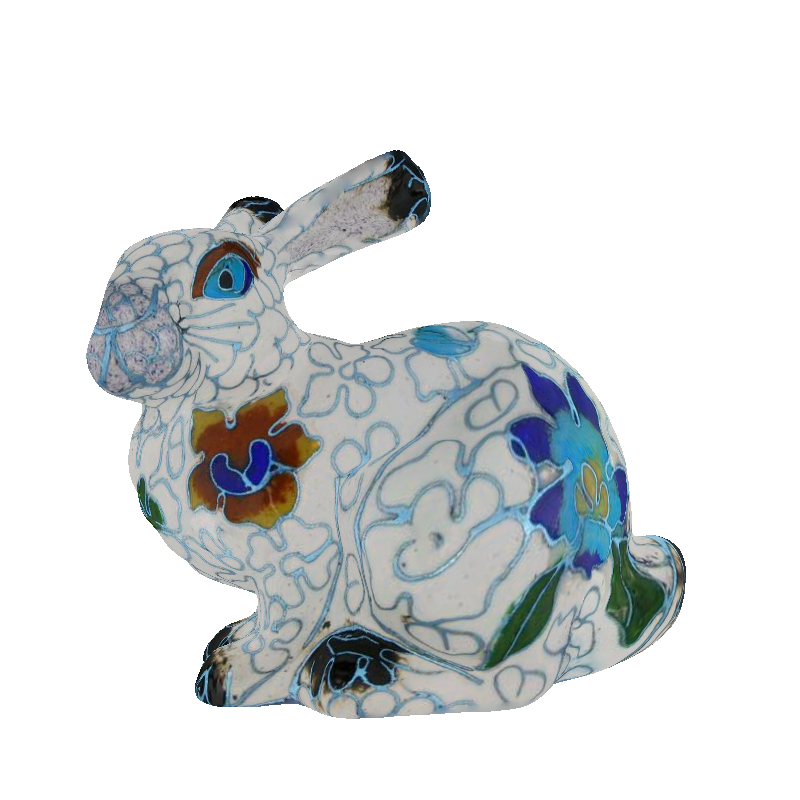

Supplement: Supplemental Information 1 [file peerj-cs-09-1584-s001.zip › py_2dto3d/video0/275.png]

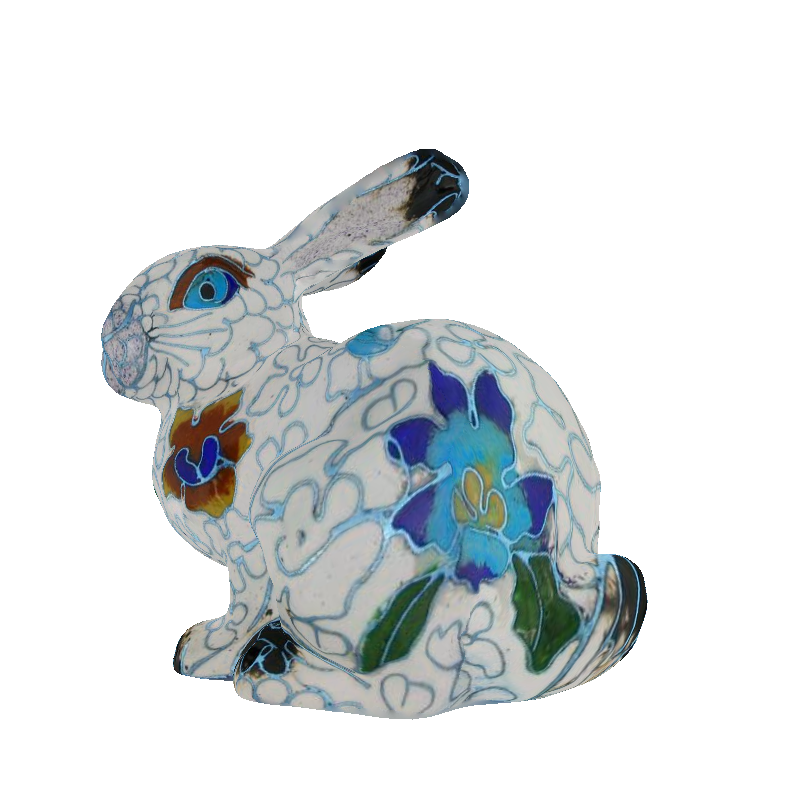

Supplement: Supplemental Information 1 [file peerj-cs-09-1584-s001.zip › py_2dto3d/video0/305.png]

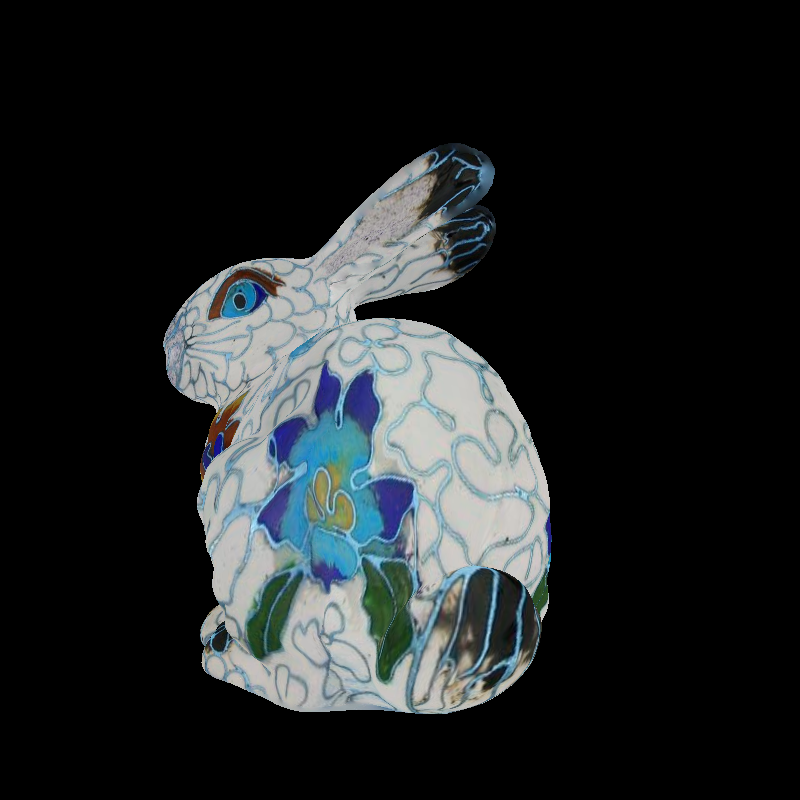

Supplement: Supplemental Information 1 [file peerj-cs-09-1584-s001.zip › py_2dto3d/video0/335.png]

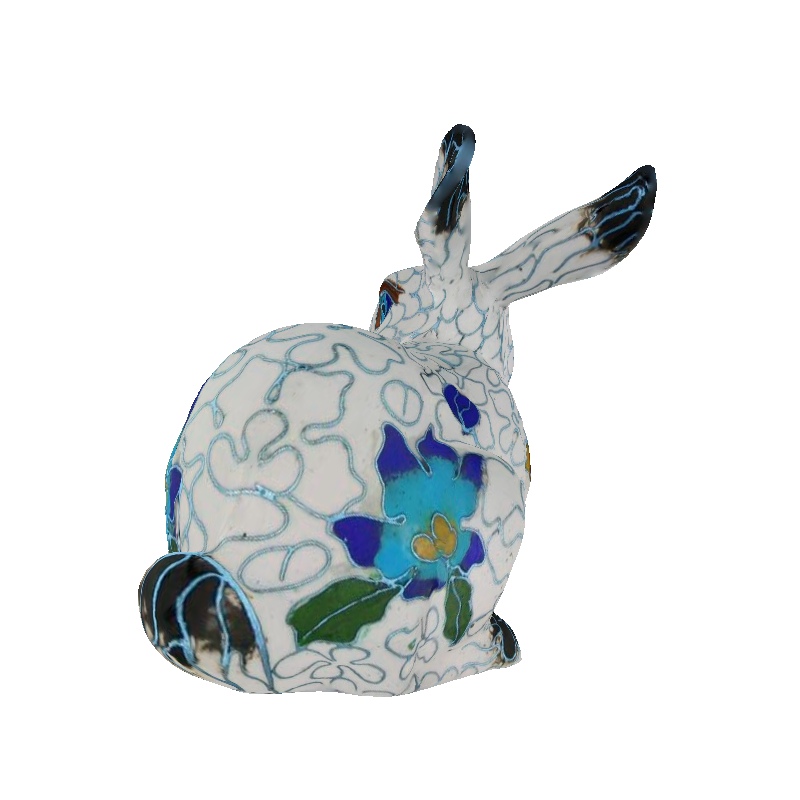

Supplement: Supplemental Information 1 [file peerj-cs-09-1584-s001.zip › py_2dto3d/video0/35.png]

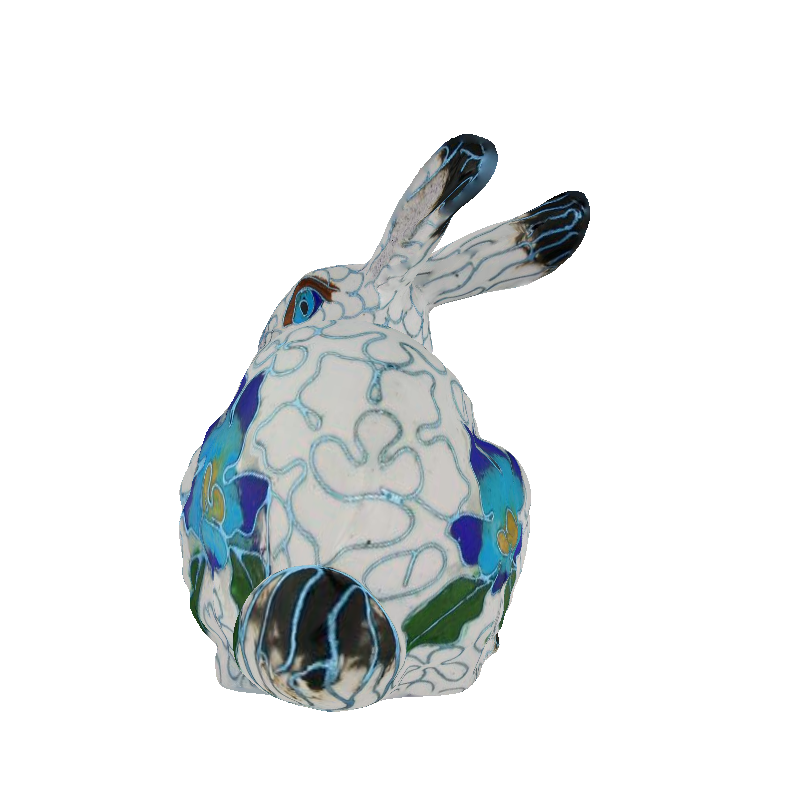

Supplement: Supplemental Information 1 [file peerj-cs-09-1584-s001.zip › py_2dto3d/video0/5.png]

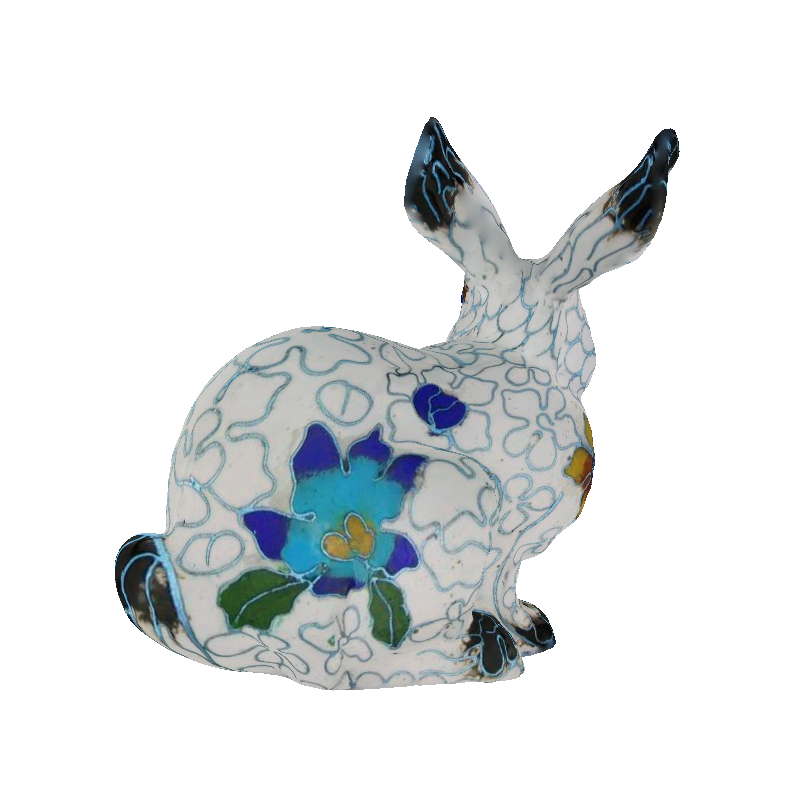

Supplement: Supplemental Information 1 [file peerj-cs-09-1584-s001.zip › py_2dto3d/video0/65.png]

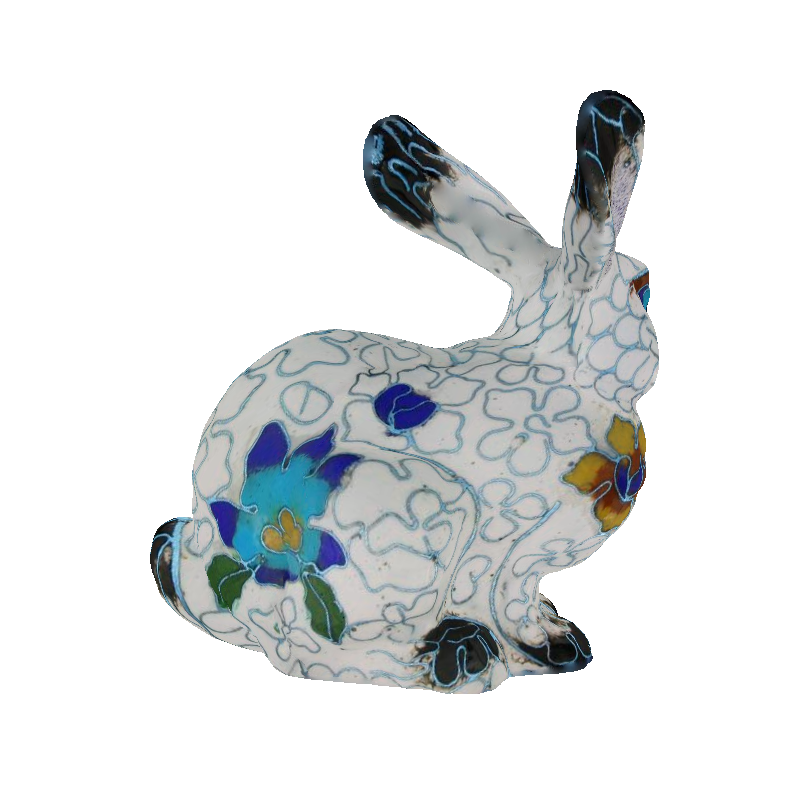

Supplement: Supplemental Information 1 [file peerj-cs-09-1584-s001.zip › py_2dto3d/video0/95.png]

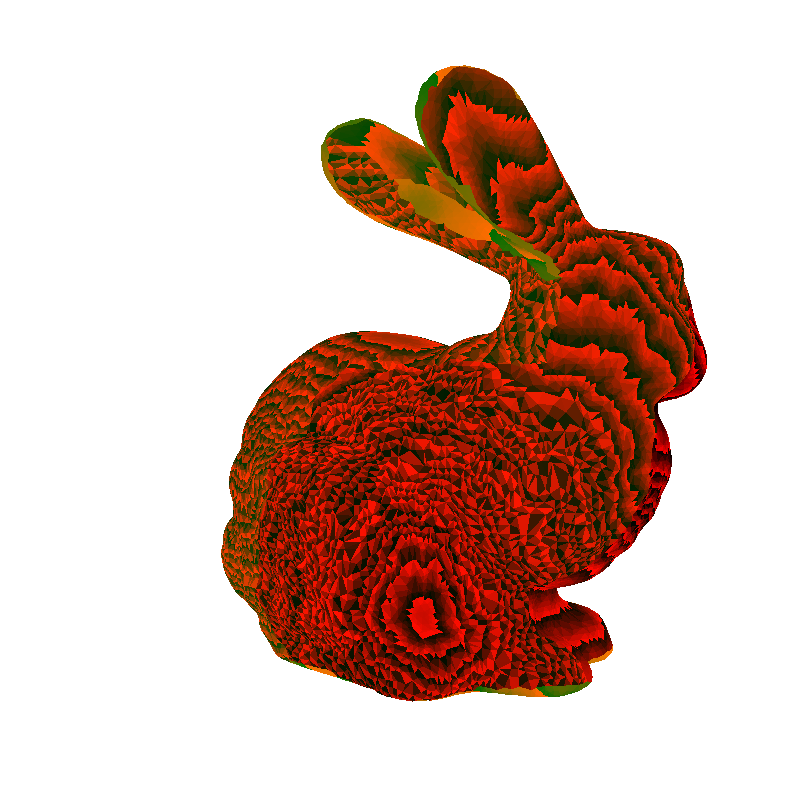

Supplement: Supplemental Information 1 [file peerj-cs-09-1584-s001.zip › py_2dto3d/video1/125.png]

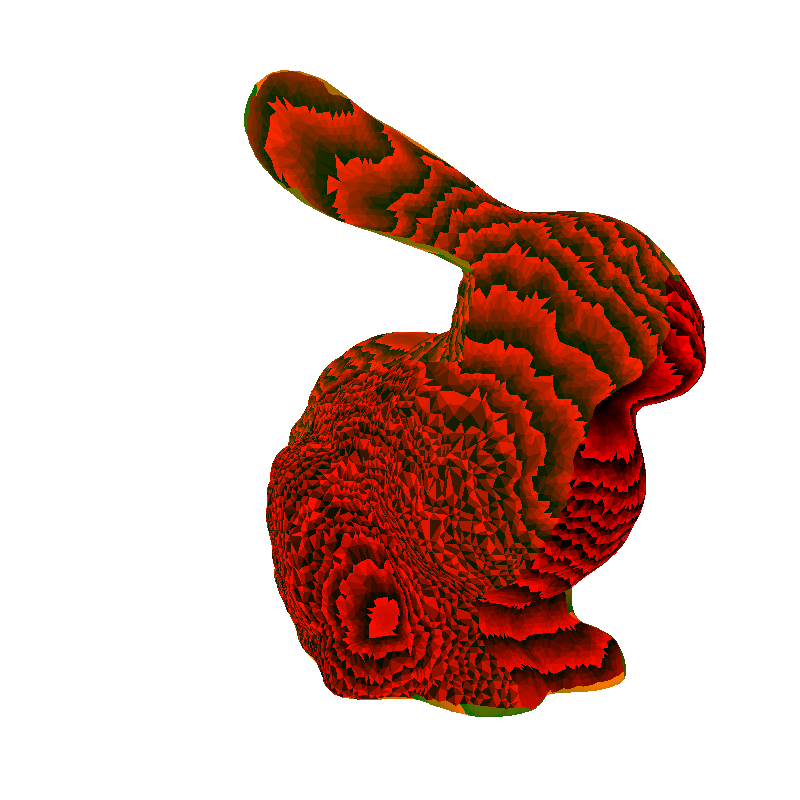

Supplement: Supplemental Information 1 [file peerj-cs-09-1584-s001.zip › py_2dto3d/video1/155.png]

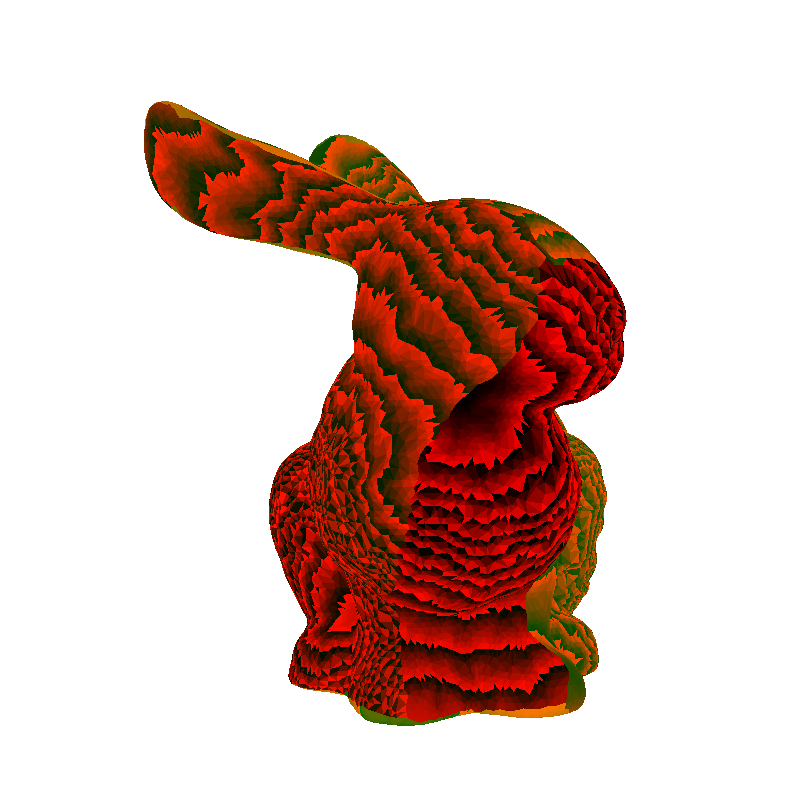

Supplement: Supplemental Information 1 [file peerj-cs-09-1584-s001.zip › py_2dto3d/video1/185.png]

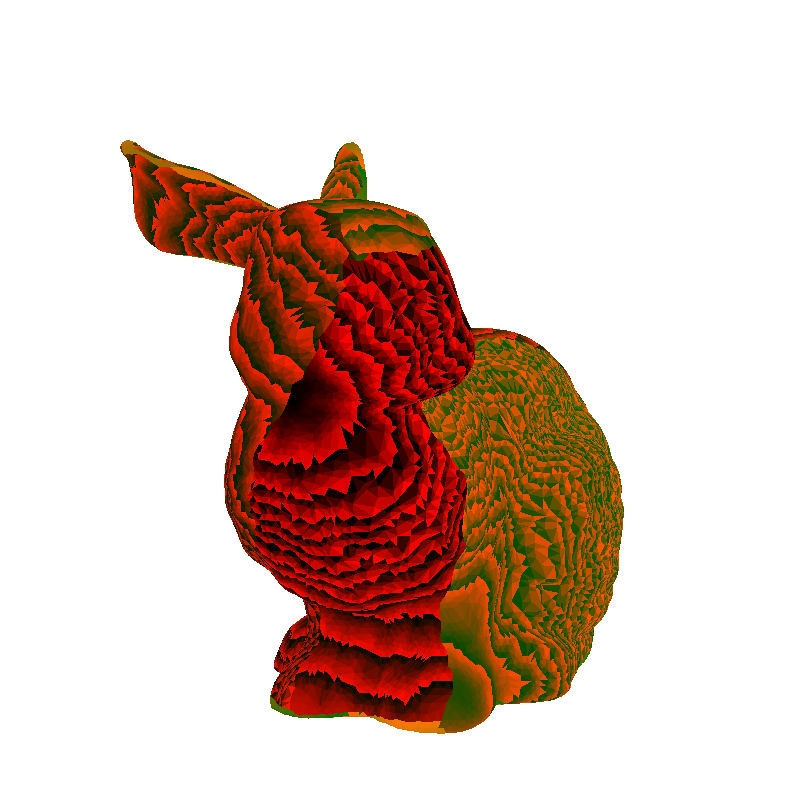

Supplement: Supplemental Information 1 [file peerj-cs-09-1584-s001.zip › py_2dto3d/video1/215.png]

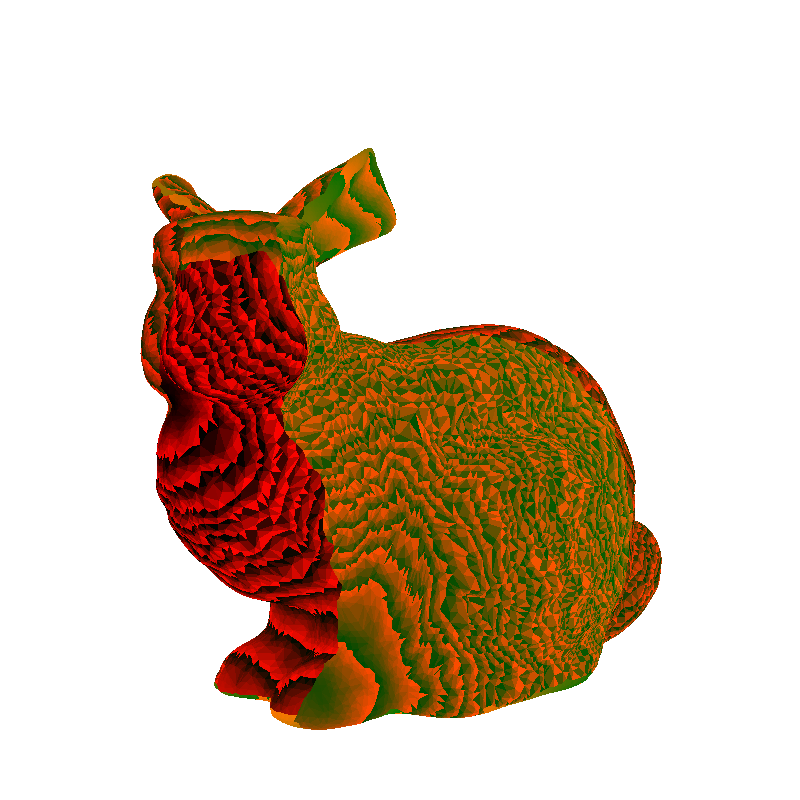

Supplement: Supplemental Information 1 [file peerj-cs-09-1584-s001.zip › py_2dto3d/video1/245.png]

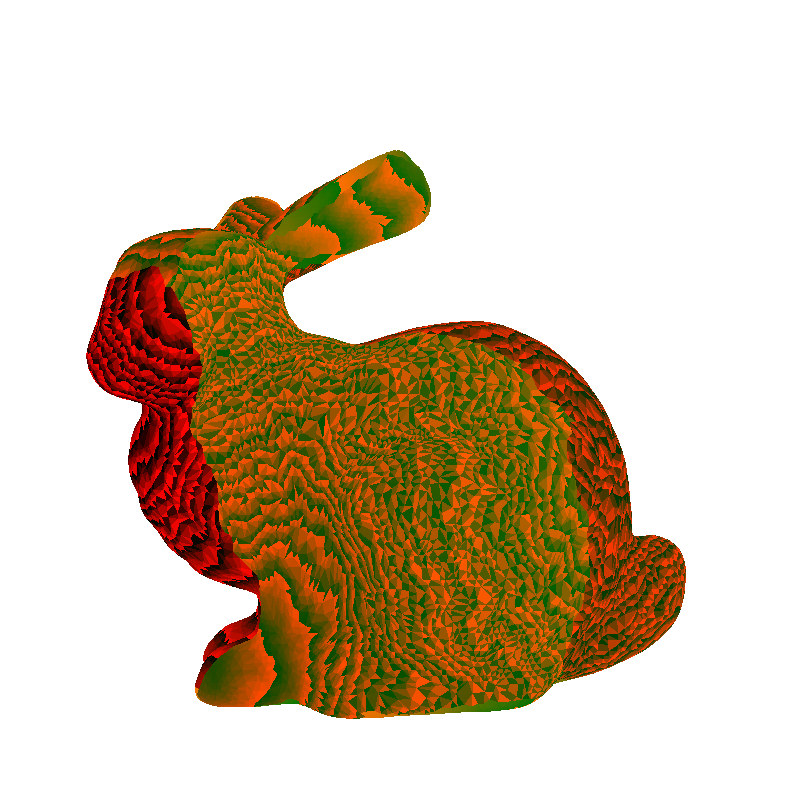

Supplement: Supplemental Information 1 [file peerj-cs-09-1584-s001.zip › py_2dto3d/video1/275.png]

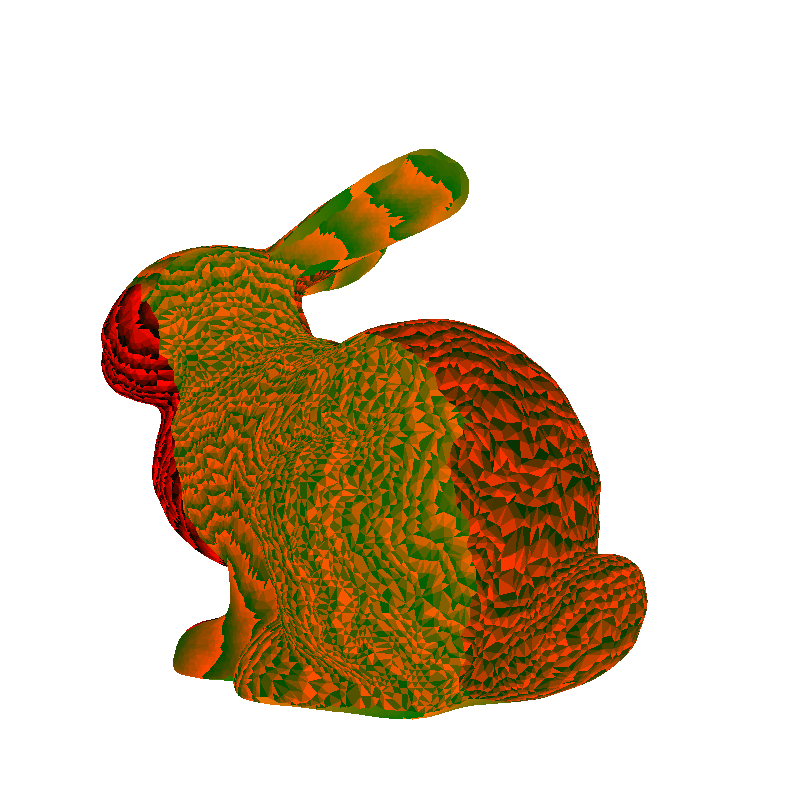

Supplement: Supplemental Information 1 [file peerj-cs-09-1584-s001.zip › py_2dto3d/video1/305.png]

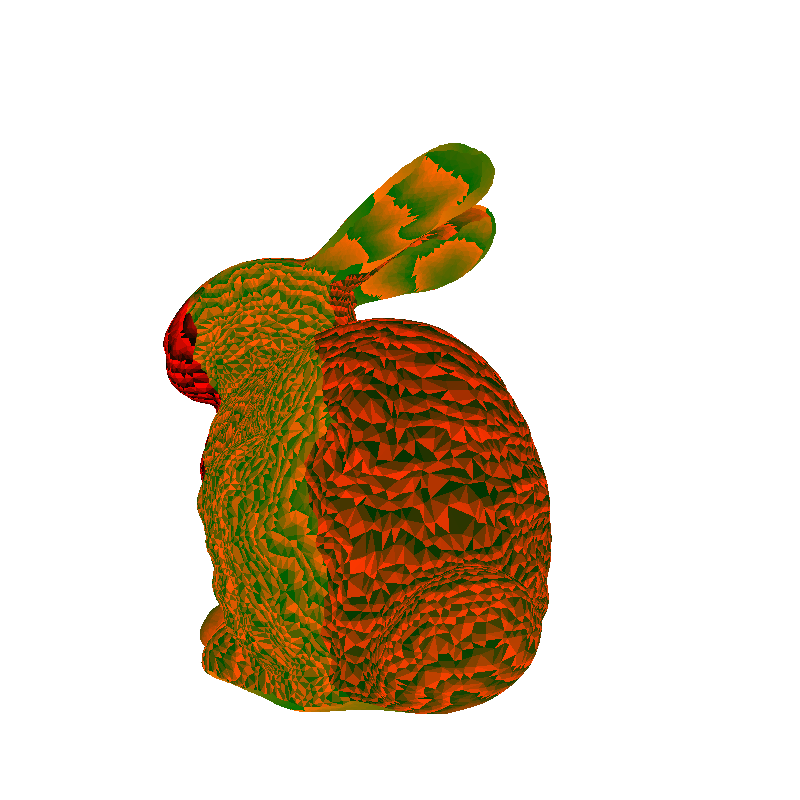

Supplement: Supplemental Information 1 [file peerj-cs-09-1584-s001.zip › py_2dto3d/video1/335.png]

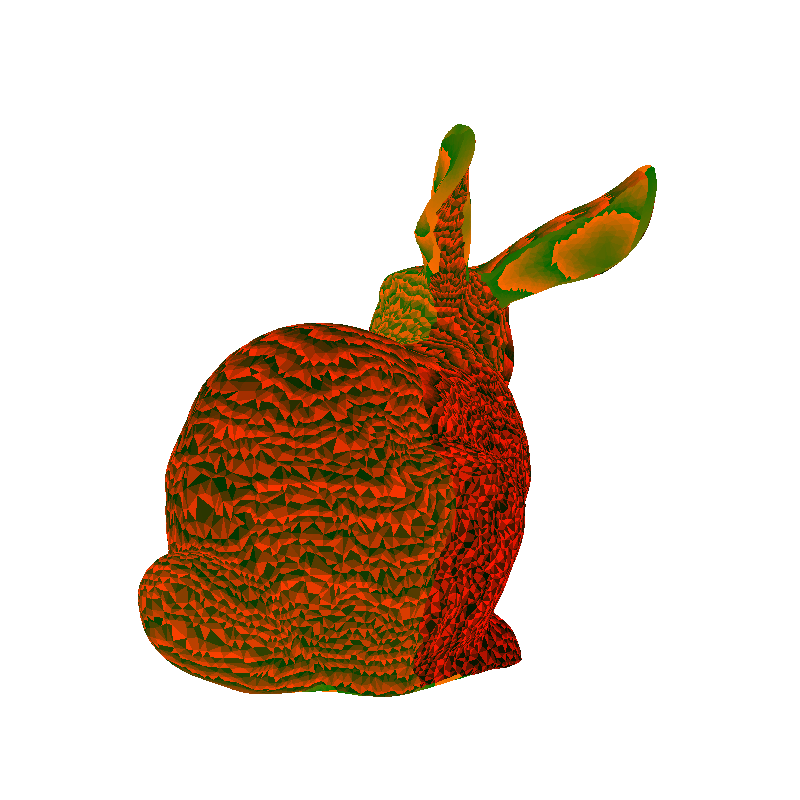

Supplement: Supplemental Information 1 [file peerj-cs-09-1584-s001.zip › py_2dto3d/video1/35.png]

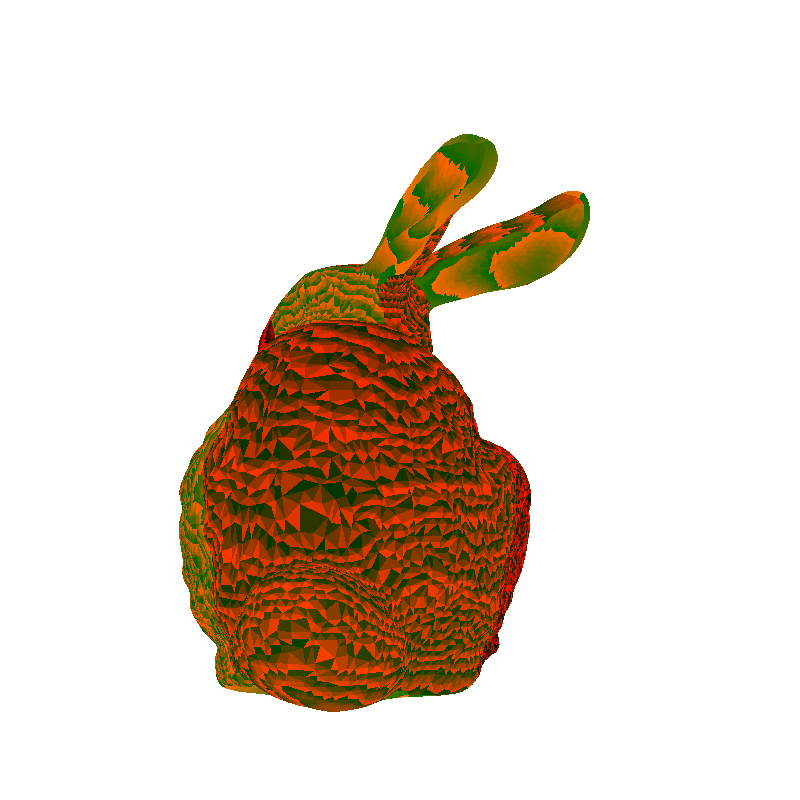

Supplement: Supplemental Information 1 [file peerj-cs-09-1584-s001.zip › py_2dto3d/video1/5.png]

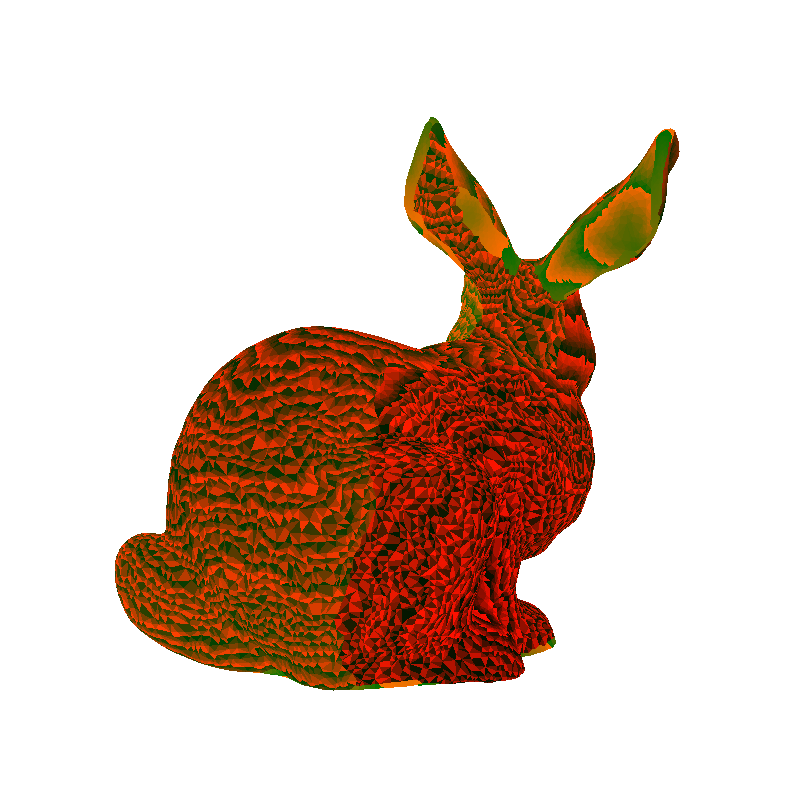

Supplement: Supplemental Information 1 [file peerj-cs-09-1584-s001.zip › py_2dto3d/video1/65.png]

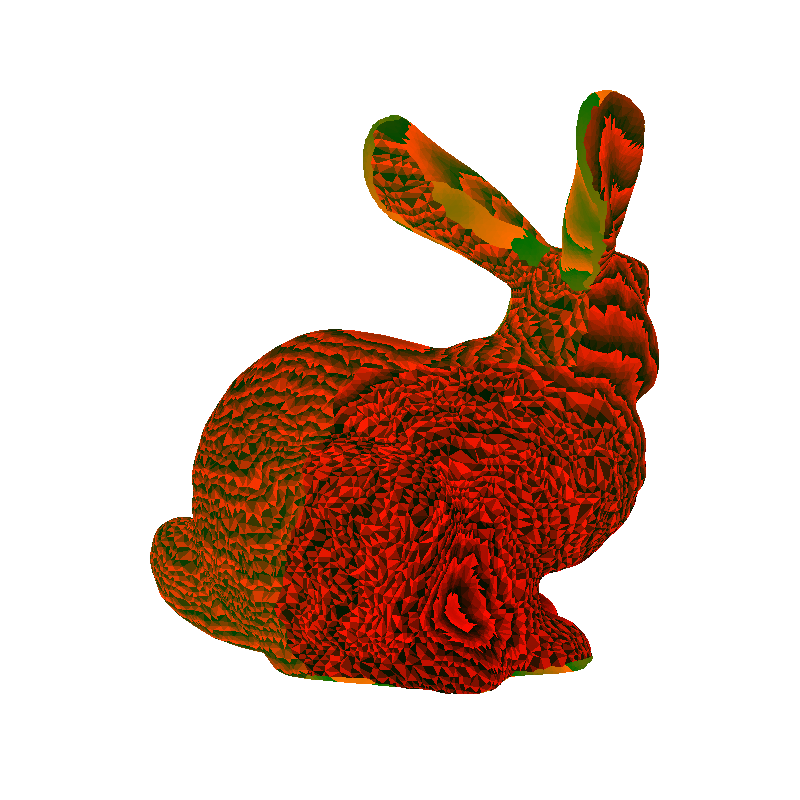

Supplement: Supplemental Information 1 [file peerj-cs-09-1584-s001.zip › py_2dto3d/video1/95.png]

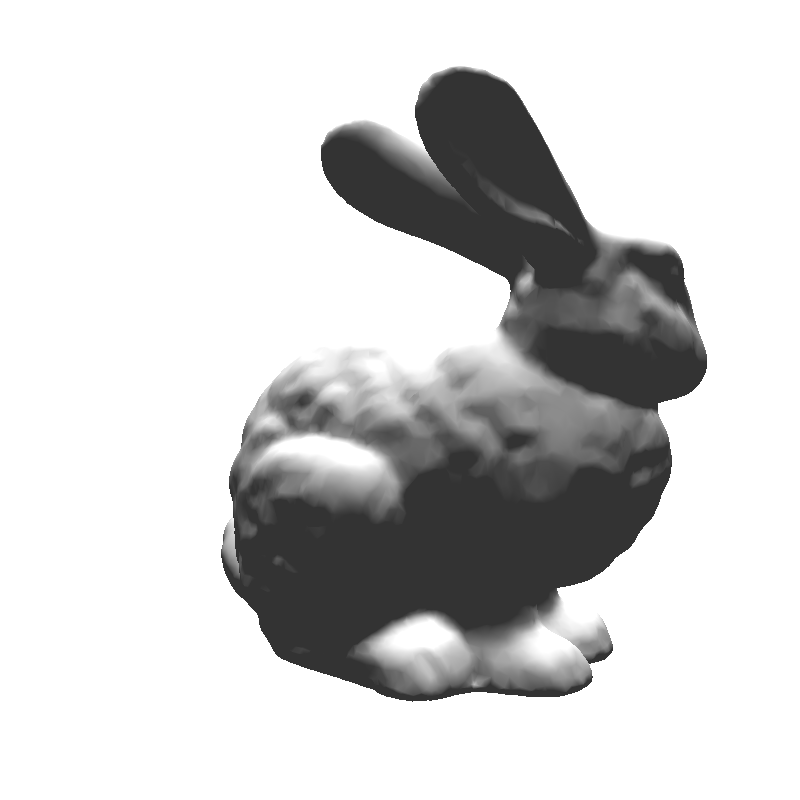

Supplement: Supplemental Information 1 [file peerj-cs-09-1584-s001.zip › py_2dto3d/video2/125.png]

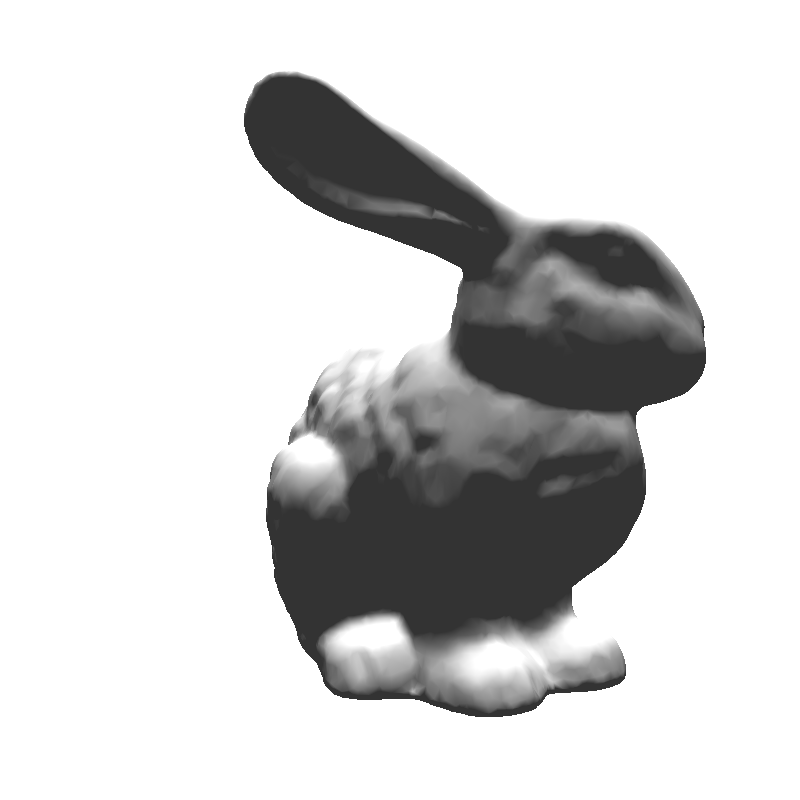

Supplement: Supplemental Information 1 [file peerj-cs-09-1584-s001.zip › py_2dto3d/video2/155.png]

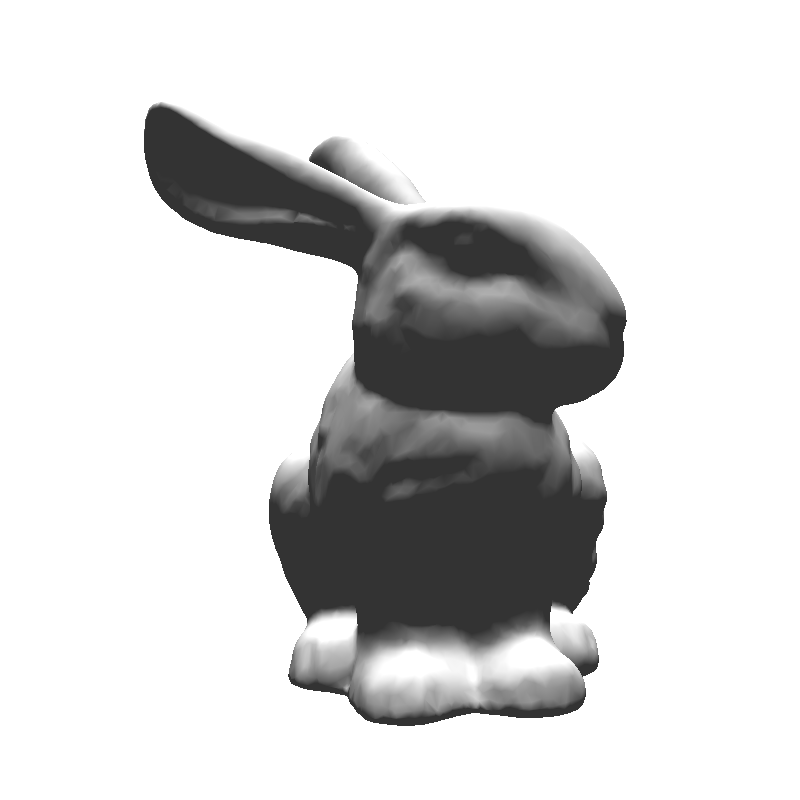

Supplement: Supplemental Information 1 [file peerj-cs-09-1584-s001.zip › py_2dto3d/video2/185.png]

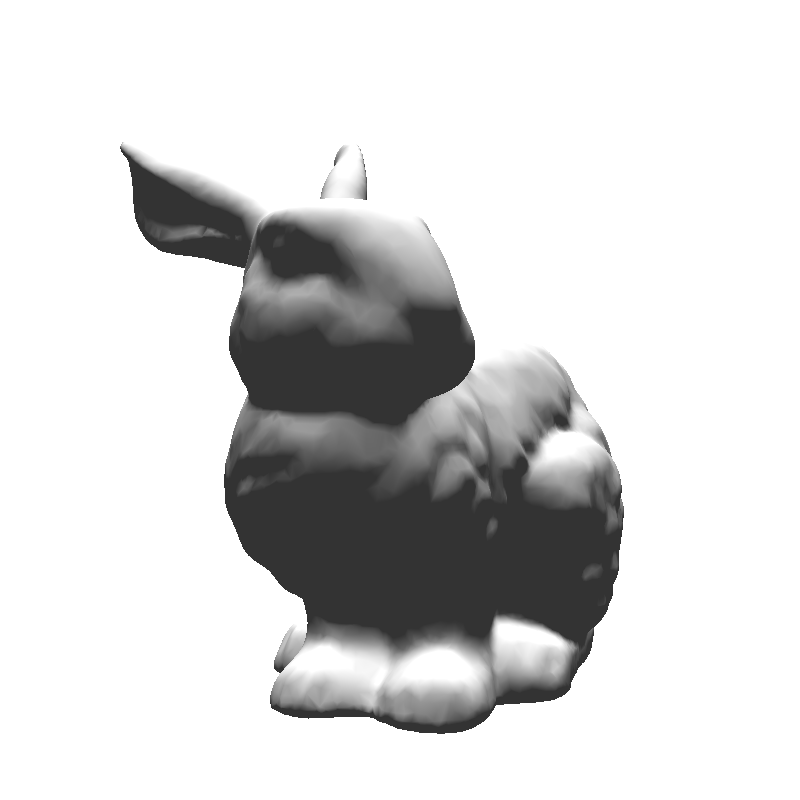

Supplement: Supplemental Information 1 [file peerj-cs-09-1584-s001.zip › py_2dto3d/video2/215.png]

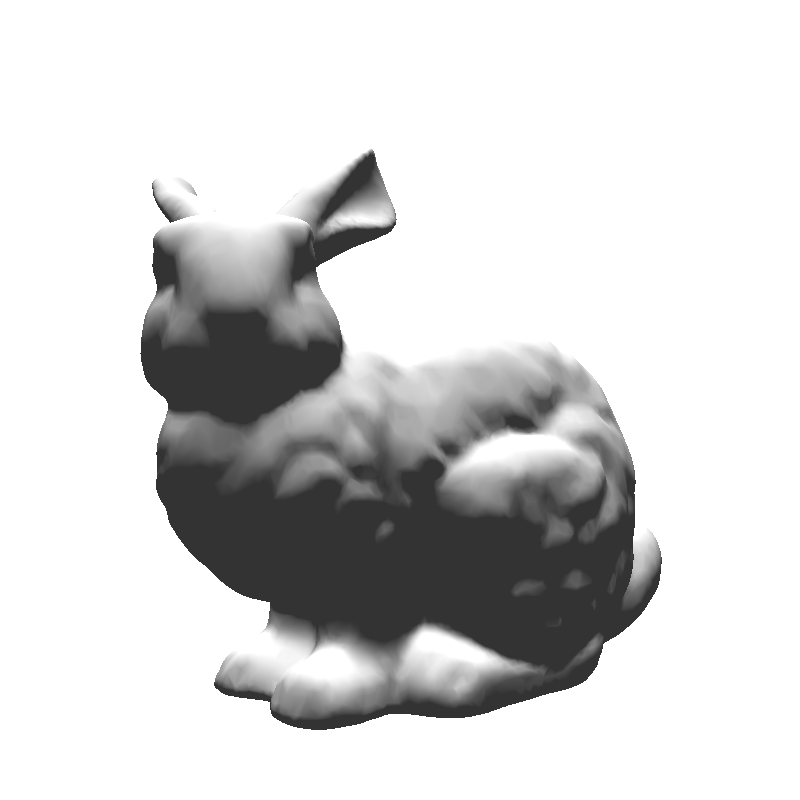

Supplement: Supplemental Information 1 [file peerj-cs-09-1584-s001.zip › py_2dto3d/video2/245.png]

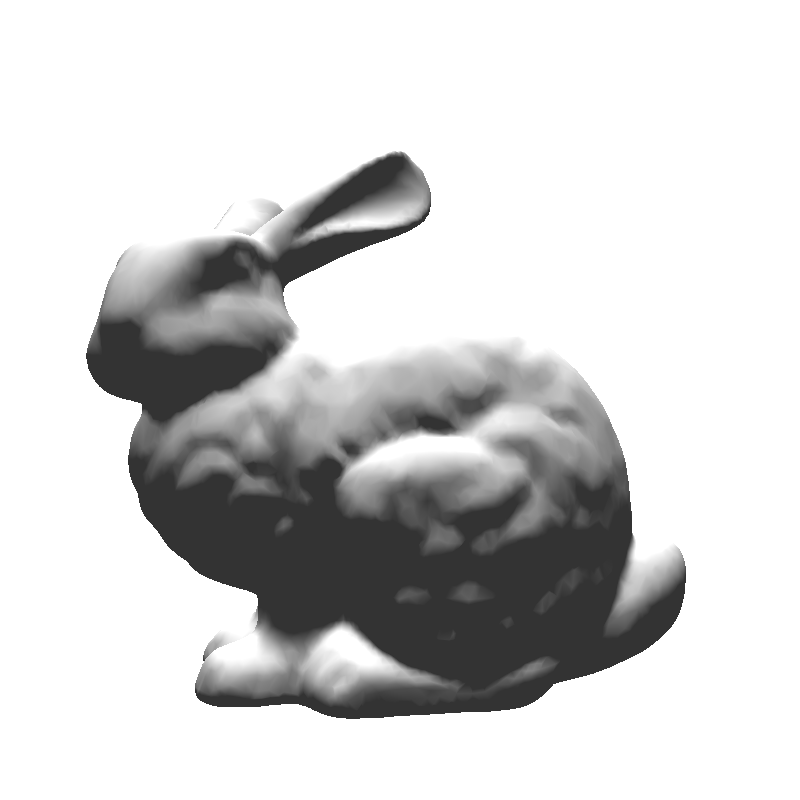

Supplement: Supplemental Information 1 [file peerj-cs-09-1584-s001.zip › py_2dto3d/video2/275.png]

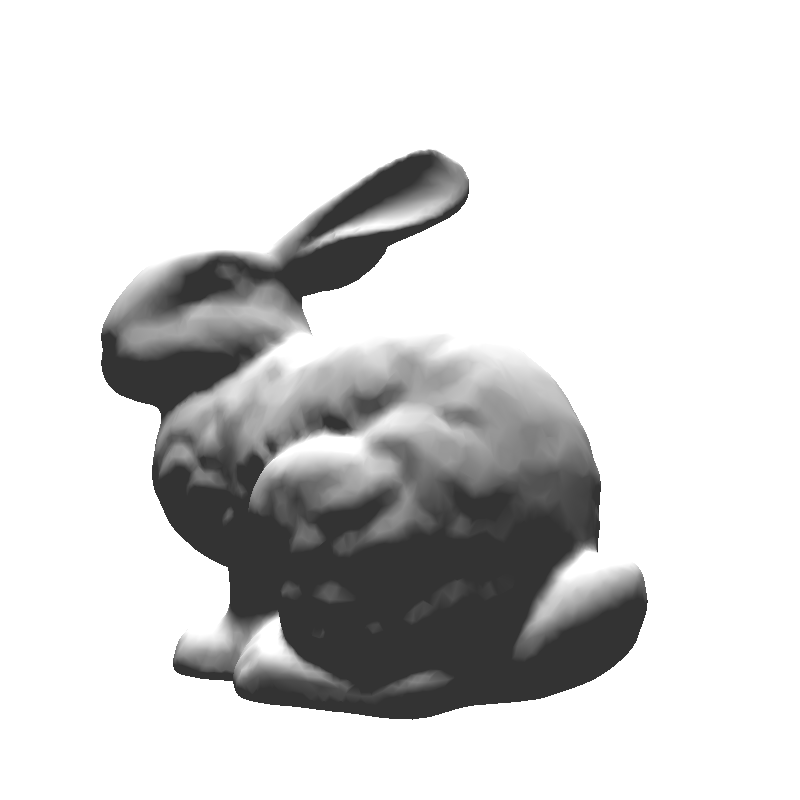

Supplement: Supplemental Information 1 [file peerj-cs-09-1584-s001.zip › py_2dto3d/video2/305.png]

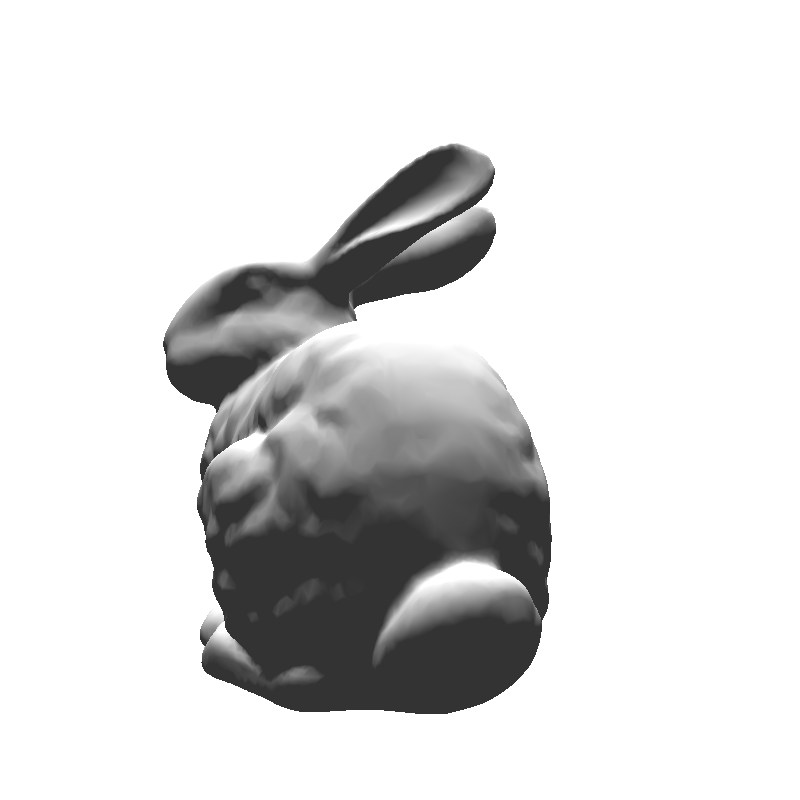

Supplement: Supplemental Information 1 [file peerj-cs-09-1584-s001.zip › py_2dto3d/video2/335.png]

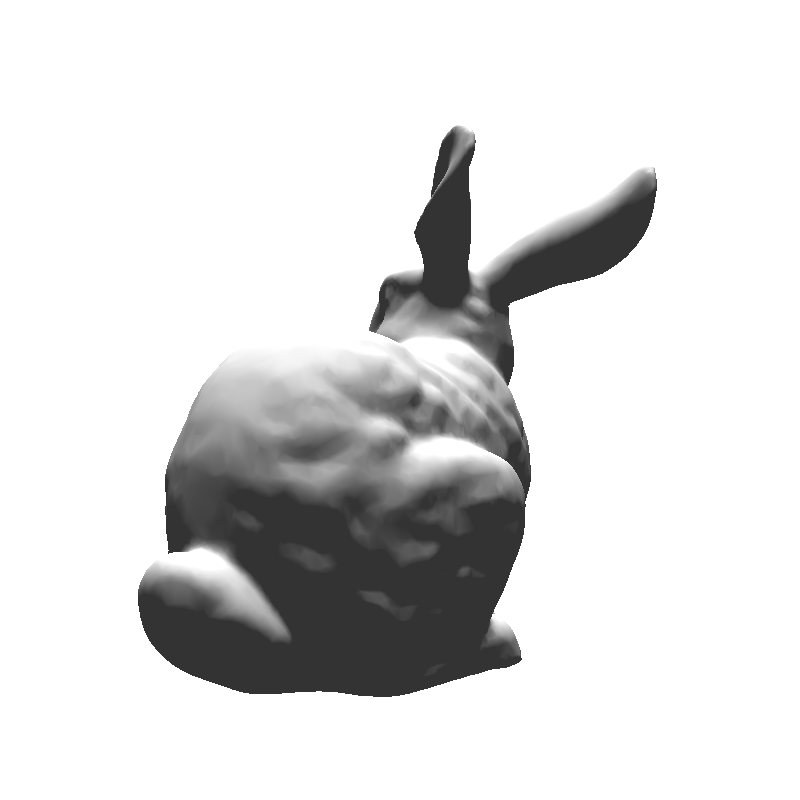

Supplement: Supplemental Information 1 [file peerj-cs-09-1584-s001.zip › py_2dto3d/video2/35.png]

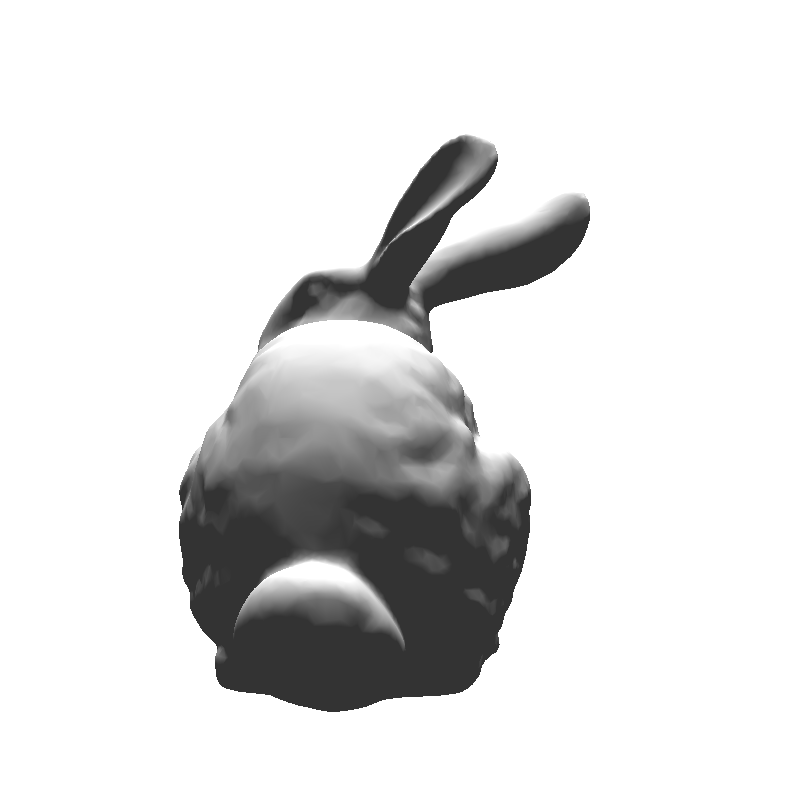

Supplement: Supplemental Information 1 [file peerj-cs-09-1584-s001.zip › py_2dto3d/video2/5.png]

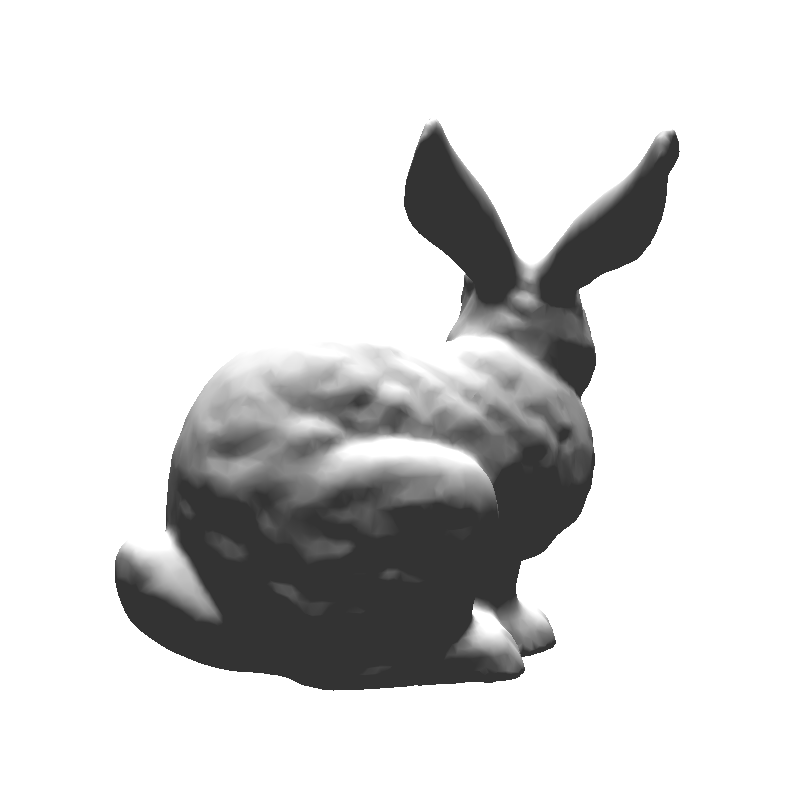

Supplement: Supplemental Information 1 [file peerj-cs-09-1584-s001.zip › py_2dto3d/video2/65.png]

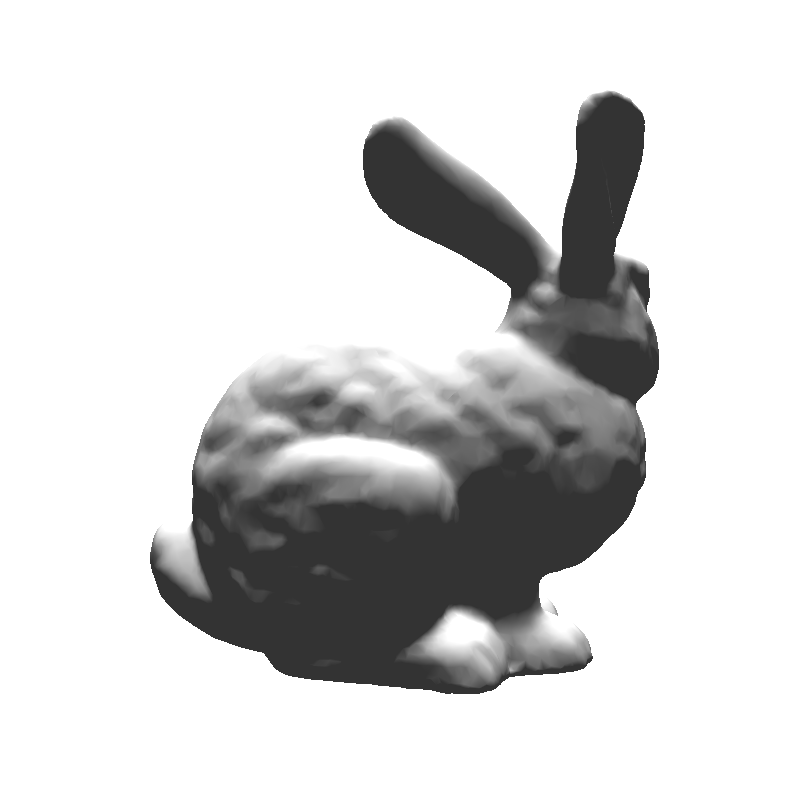

Supplement: Supplemental Information 1 [file peerj-cs-09-1584-s001.zip › py_2dto3d/video2/95.png]
